# Mediterranean diet and physical functioning trajectories in Eastern Europe: Findings from the HAPIEE study

**DOI:** 10.1371/journal.pone.0200460

**Published:** 2018-07-12

**Authors:** Denes Stefler, Yaoyue Hu, Sofia Malyutina, Andrzej Pajak, Ruzena Kubinova, Anne Peasey, Hynek Pikhart, Fernando Rodriguez-Artalejo, Martin Bobak

**Affiliations:** 1 Department of Epidemiology and Public Health, University College London, London, United Kingdom; 2 Max Planck Institute for Demographic Research, Rostock, Germany; 3 Institute of Internal and Preventive Medicine, Siberian Branch of the Russian Academy of Medical Sciences, Novosibirsk, Russia; 4 Department of Epidemiology and Population Studies, Jagiellonian University Collegium Medicum, Krakow, Poland; 5 National Institute of Public Health, Prague, Czech Republic; 6 Department of Preventive Medicine and Public Health, School of Medicine, Universidad Autónoma de Madrid/IdiPaz and CIBERESP, Madrid, Spain; CUNY, UNITED STATES

## Abstract

**Background:**

Unhealthy diet may increase the risk of impaired physical functioning in older age. Although poor diet and limited physical functioning both seem to be particularly common in Eastern Europe, no previous study has assessed the relationship between these two factors in this region. The current analysis examined the association between overall diet quality and physical functioning in Eastern European populations.

**Methods:**

We used data on 25,504 persons (aged 45–69 years at baseline) who participated in the Health Alcohol and Psychosocial factors in Eastern Europe (HAPIEE) study. Dietary assessment at baseline used food frequency questionnaire, and the overall diet quality was evaluated by the Mediterranean diet score (MDS). Physical functioning (PF) was measured by the physical functioning subscale (PF-10) of the 36-item Short-Form Health Survey at baseline and three subsequent occasions over a 10-year period. The cross-sectional and longitudinal relationships between the MDS and PF were examined simultaneously using growth curve models.

**Results:**

Men and women with higher adherence to the Mediterranean diet had significantly better PF at baseline; after multivariable adjustment, the regression coefficient per 1-unit increase in the MDS was 0.39 (95% CI: 0.25, 0.52) in men and 0.50 (0.36, 0.64) in women. However, we found no statistically significant link between baseline MDS and the subsequent slope of PF decline in neither gender; the coefficients were -0.02 (-0.04, 0.00) in men and -0.01 (-0.03, 0.02) in women.

**Discussion:**

Our results do not support the hypothesis that the Mediterranean diet has a substantial impact on the trajectories of physical functioning, although the differences existing at baseline may be related to dietary habits in earlier life.

## Introduction

Good physical functioning is one of the most important criteria for healthy aging [1]. Similarly to many other health indicators in older age, the level of physical functioning and the rate of its decline are modifiable by lifestyle factors [2,3]. For example, previous research has shown that dietary factors, including intakes of fruits and vegetables, dairy products and various micronutrients, are related to physical functioning limitations, disability or sarcopenia [3–8].

Given that individuals do not eat nutrients but meals with complex combinations of foods which may interact, overall dietary patterns provide a holistic approach to study the relationship between diet and health [9–11]. They also help to overcome some of the limitations of the traditional focus on individual foods or nutrients [12]. One pattern which has shown protective effect against diverse diseases in both observational and interventional studies is the Mediterranean dietary pattern [13,14]. Several studies have reported inverse relationships between Mediterranean diet and the rate of cognitive decline [15,16], but the evidence on physical functioning is relatively sparse. Those analyses which did examine this link found mostly significant associations, indicating increased mobility, muscle strength and lower rate of physical decline in individuals whose eating habits follow closely the Mediterranean diet [17–20]. A recent systematic review also found that Mediterranean diet reduces the risk of frailty in community-dwelling elderly [21].

Unhealthy diet has also been suggested as one of the main reasons for poor health in Eastern Europe [22–24], a region where the prevalence of impaired physical functioning is likely to be higher than in Western Europe [25]. To date, the association between diet quality and physical functioning in Eastern European populations has not been examined. Although the extent to which the Mediterranean diet can be adapted to non-Mediterranean countries needs further assessment [26], the beneficial effects of this dietary pattern on mortality in Eastern Europeans have been reported [27]. The aim of the current analysis was to assess the association of Mediterranean diet with the level and trajectories of physical functioning in three Eastern European populations participating in the Health Alcohol and Psychosocial factors in Eastern Europe (HAPIEE) cohort study.

## Methods

### Study population and analytical sample

The HAPIEE study was approved by ethics committees at University College London, UK, and all local centres in the Czech Republic, Poland and Russia (The Joint UCL/UCLH Committees on the Ethics of Human Research (Committee Alpha), London, UK; Ethical Committee of the Institute of Internal Medicine, Siberian Branch of the RAMS, Novosibirsk, Russia; Ethical Commission of the National Institute of Public Health, Prague, Czech Republic Ethical Commission of the Jagellonian University Collegium Medicum, Krakow, Poland). All participants gave informed consent.

The HAPIEE cohort was established in 2002–05. Random samples of men and women aged 45–69 years, stratified by sex and 5-year age groups, in Novosibirsk (Russia), Krakow (Poland) and seven middle-sized towns in the Czech Republic (Havířov/Karviná, Jihlava, Ústí nad Labem, Liberec, Hradec Králové, and Kromĕříz) were invited to participate. The study was approved by ethics committees at University College London and all local centres. All participants gave informed consent. A detailed description of the study is provided elsewhere [28].

From 28,783 participants, we excluded those who stated that the list of foods in the food frequency questionnaire (FFQ) did not represent their diet (n = 800), those with incomplete dietary data (more than 10% missing answers in the FFQ (n = 679)) or reported implausible energy intake (more than 5000 kcal/day or less than 800kcal/day in males; more than 4500kcal/day or less than 500kcal/day in females (n = 396)) or missing data regarding olive oil usage (n = 1404). After these exclusions, 25,504 subjects (7215 Czechs, 9042 Russians and 9247 Poles) were included in the analysis.

### Measurements

At baseline, all participants completed a structured questionnaire and underwent a short medical examination. The questionnaire was translated into local languages and back-translated into English to ensure accuracy and cross-cultural comparability, and was piloted in a separate sample [29]. Participants were re-examined in 2006–08 using Computer Assisted Personal Interview, and further follow-up in 2009 and 2012 used postal questionnaires.

Physical functioning (PF) was measured by the physical functioning subscale (PF-10) of the 36-item Short-Form Health Survey (SF-36) at baseline and subsequent follow-ups. The PF-10 evaluates limitations in ten activities: (1) vigorous activities, such as lifting heavy objects and doing strenuous sports; (2) moderate activities, such as moving a table and pushing a vacuum cleaner; (3) lifting/carrying a bag of groceries; walking (3) two kilo-meters, (4) one kilo-meter, (5) one hundred meters; climbing (6) several flights of stairs, (7) one flight of stairs; (8) bending; (9) kneeling or stooping; (10) bathing and dressing. Participants indicated the extent of which these activities were limited by their health with the option of choosing from three possible self-reported answers: limited a lot, limited a little or not limited at all. All questionnaires were administered with the help of a research nurse. A summary PF-10 score (range 0–100) was calculated, with a higher score indicating better PF [30].

Data on self-reported dietary intake at baseline was collected using a validated semi-quantitative FFQ with 136, 147 and 148 food and drink items in the Czech, Russian and Polish samples, respectively [31]. The questionnaires were self-completed in Poland and the Czech Republic, and nurse-administered in Russia. The European Food Safety Authority`s FoodEx 2 food classification and description system was used to categorise food items into food groups [32], and nutrient intake levels were calculated with the McCance and Widdowson Food Composition Database. In order to assess the participants`adherence to the Mediterranean diet, we used a scoring system which was originally developed by Sofi and colleagues [33]. This method applies pre-defined absolute cut-off values, based on a review of food intake distributions in previous Mediterranean diet studies, to calculate individual component scores for the intakes of vegetables, fruits and nuts, legumes, cereals, fish, olive oil (positive scoring), meat, dairy products (negative scoring) and alcohol (moderate intake scores highest). Previous analysis showed that the Mediterranean diet score (MDS) obtained by this approach predicted mortality in this sample. In fact, the observed associations between MDS and mortality were found to be stronger than those with the “traditional”MDS version developed by Trichopoulou [27].

In addition to age at baseline (centred on the population mean of 58 years), several baseline characteristics were included as covariates because they can be associated with both diet and physical functioning. Smoking status was coded as never, former and current smoking. Marital status was dichotomized into married/cohabiting or living alone. Participants’ socioeconomic status was assessed by their highest educational attainment (less than secondary school, secondary school, university), a sum of 12 household items ownership (e.g., microwave, dishwasher, car, holiday cottage, etc.), and economic activity (working, pensioners and still working, pensioners and not working, unemployed). Participants reported whether they had been diagnosed or hospitalized for spine or joint problems in the past year before baseline.

### Statistical analysis

Missing data was handled by multiple imputation by chained equations (MICE) [34,35]. A number of variables at baseline were used as auxiliary variables in the imputation, including self-rated health, long-term health problem, history of cardiovascular disease, hypertension, cancer, injury, physical activity, number of household amenities at age ten, depressive symptoms, and frequency of contacting relatives and friends. Specific to Czechs and Poles, attendance to the medical examination at baseline was also used as it predicted the PF-10 scores. During the follow-up, 791 Czech (9.0%), 1,109 Russian (11.9%) and 830 Polish (7.8%) participants died. Compared with survivors, deceased individuals disproportionally contained those from lower SES groups, with poorer health status and less favourable health behaviours, and those with poor baseline PF and faster decline in PF (selective mortality). To take this into account, we also imputed missing PF-10 scores for participants who died during follow up. A total of 70 imputed datasets were generated in Stata 12 (StataCorp, 2013) as the number of imputed datasets should be equal or greater than the proportion of incomplete cases (cases with missing value in at least one study variable) [35]. Missing follow-up years due to non-response during follow-up were replaced by a random number generated under a normal distribution of observed follow-up years.

Individual trajectories of the PF-10 score during the follow-up in the multiply imputed datasets were estimated by latent growth curve modelling in Mplus 6 (Muthén & Muthén, 1998–2011) [36–38]. Visual inspection indicated a linear decline in the PF-10 score over the follow-up years in all cohorts, thus a linear growth curve model was used in this study. Two latent growth factors describe the linear PF-10 trajectories: the initial status of the score at baseline and the rate of change in the score per year of follow-up (slope). The relationships between the MDS and physical functioning were assessed both cross-sectionally using baseline dietary and PF-10 data (initial status at baseline), and longitudinally in terms of change in physical functioning (slope). Two models were used in the analyses. The first model was adjusted for baseline age and country cohort (in case of the pooled analysis); the second model was further adjusted for marital status, education, ownership of household items, economic activity, history of spine/joint problems, and smoking status. MDS was used as a categorical as well as a continuous variable. In the categorical analysis participants`adherence to the Mediterranean diet was classified as low (MDS score 1–7), moderate (8–10) or high (11–16), using the “low” category as reference. Results are presented separately by gender and country cohort.

The age trend of PF-10 score at baseline and its decline during follow-up by MDS category were illustrated with ageing-vector graphs [39,40]. All graphs were based on results from model 2 and produced in Stata 12.

## Results

Table 1 shows the baseline characteristics of the Czech, Russian and Polish cohorts. Due to the stratified sampling method the distribution of participants across the 5-year age groups was balanced in all three cohorts. High adherence to the Mediterranean diet was found in 30% of Czechs and Poles and in less than 15% amongst Russians.

**Table 1:** Characteristics of the analytical sample at baseline.

	Country cohorts		
Covariates	Czech Republic	Russia	Poland
	(n = 7215)	(n = 9042)	(n = 9247)
**Age** (years, %)			
45–49	17.4	16.9	18.4
50–54	20.2	19.4	20.6
55–59	19.2	21.6	21.1
60–64	22.6	19.1	19.9
65–69	20.6	23.0	20.0
**Sex** (%)			
Male	46.6	45.1	48.3
Female	53.4	54.9	51.7
**Marital status (%)**			
Married/cohabiting	75.9	72.2	76.8
Single/divorced/widowed	24.1	27.8	23.2
**Education** (%)			
Less than secondary education	48.1	37.1	32.7
Secondary education	37.7	33.9	38.5
University	14.3	29.0	28.8
**Ownership of household items** (sum of 12 items)			
Mean	6.9	5.7	6.4
SD	2.3	2.1	2.2
**Economic activity** (%)			
Working	46.5	35.9	38.4
Pensioner still working	7.9	18.5	6.5
Pensioner not working	42.7	41.6	40.1
Unemployed	2.9	4.0	5.0
**Spine/joint problems** (%)			
No	44.6	34.3	30.3
Yes, not hospitalised	42.8	56.3	61.5
Yes, hospitalised	12.5	9.4	8.2
**Smoking** (%)			
Never	43.6	58.5	40.4
Former	29.8	13.5	28.3
Current	26.5	28.0	31.3
**Mediterranean diet score** (%)			
Low (1–7)	23.7	35.2	19.2
Moderate (8–10)	46.2	50.3	50.2
High (11–16)	30.1	14.5	30.6

Changes of the mean PF-10 scores over time in the three country cohorts are presented in Table 2 and in S1 and S2 Figs. Mean PF-10 score decreased over the 10-year observational period in all three cohorts and both genders. In men, the baseline score was similar across the cohorts but the decline in physical functioning was significantly steeper in Russians (decline in PF-10 score per year: -2.03 (95%CI: -2.23; -1.84)) and Poles (-1.56 (-1.67; -1.44)) than for the Czechs (-0.74 (-0.85; -0.62)). In women both the initial score and the overall slope indicated significantly better physical functioning in the Czech sample compared to the other two cohorts. Country heterogeneity of both the intercept and slope were statistically significant (p<0.001).

**Table 2:** Mean PF-10 scores at each assessment occasions in the three country cohorts.

Sex	Data collection periods	Czech Republic		Russia		Poland		Pooled	
		Mean	(SD)	Mean	(SD)	Mean	(SD)	Mean	(SD)
MALES	Baseline (2002–2005)	85.28	(18.00)	86.87	(18.41)	84.28	(19.81)	85.45	(18.87)
	Re-examination (2006–2008)	83.75	(16.94)	84.61	(20.67)	77.13	(20.17)	81.56	(19.80)
	Postal Questionnaire (2009)	80.34	(22.42)	75.15	(26.77)	75.55	(25.89)	76.77	(25.38)
	Postal Questionnaire (2012)	78.79	(23.61)	69.16	(29.56)	70.38	(26.40)	72.34	(27.11)
	Slope (coefficient, 95%CI)^a^	-0.74 (-0.85, -0.62)		-2.03 (-2.23, -1.84)		-1.56 (-1.67, -1.44)		-1.49 (-1.58, -1.40)	
FEMALES	Baseline (2002–2005)	82.37	(18.91)	77.42	(21.16)	77.24	(21.77)	78.76	(20.90)
	Re-examination (2006–2008)	81.20	(18.28)	75.38	(22.95)	71.35	(21.65)	75.61	(21.61)
	Postal Questionnaire (2009)	78.42	(22.44)	63.36	(27.08)	66.56	(27.14)	68.75	(26.61)
	Postal Questionnaire (2012)	77.53	(23.54)	59.39	(28.93)	61.58	(27.70)	65.30	(28.15)
	Slope (coefficient, 95%CI)^a^	-0.59 (-0.68, -0.50)		-2.18 (-2.30, -2.06)		-1.75 (-1.85, -1.64)		-1.57 (-1.63, -1.50)	

^a^Adjusted for age at baseline (age was centered at the population mean of 58 years old)

**Fig 1:**
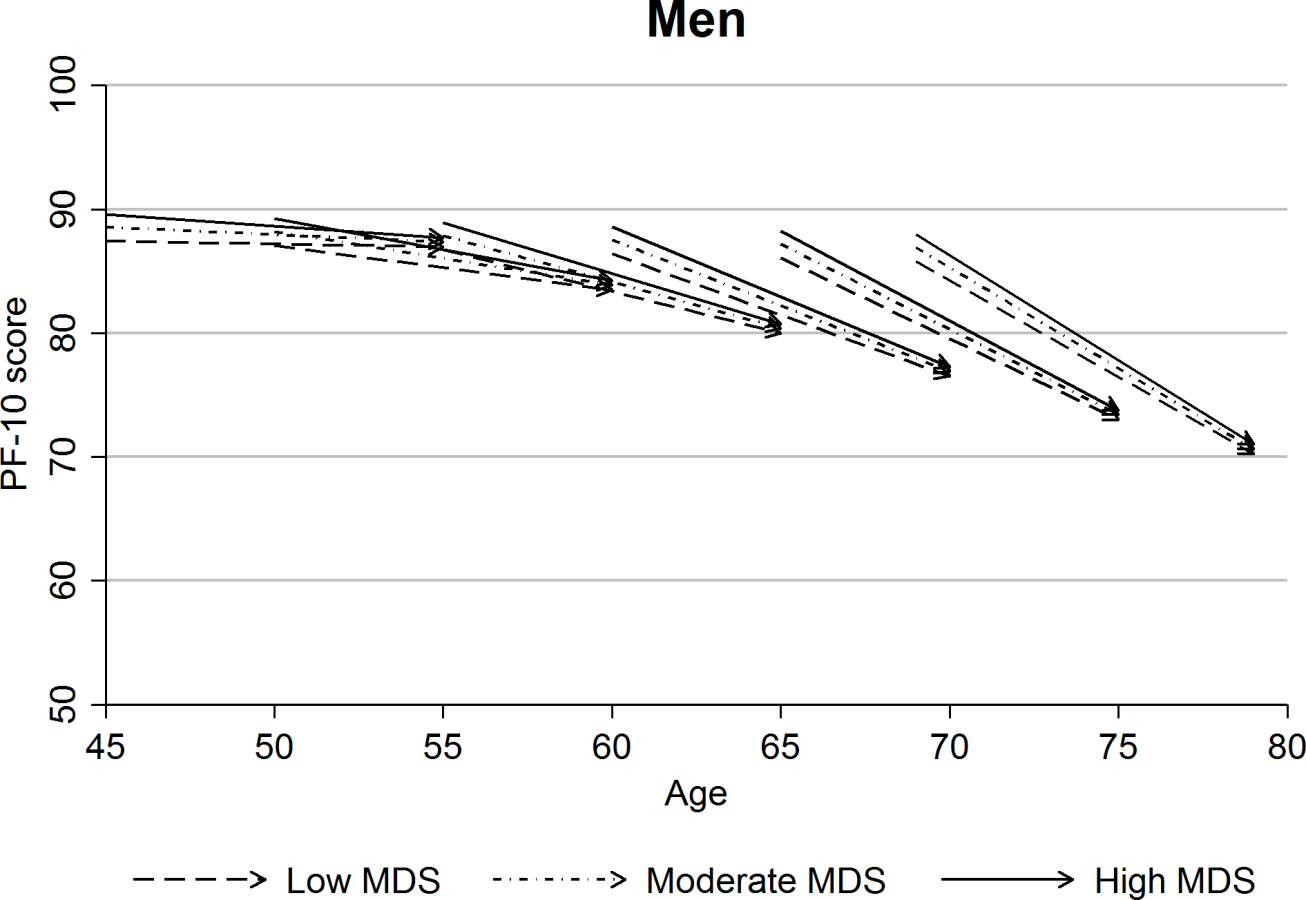
Ageing-vector graphs of predicted initial status and slope of PF-10 score during the 10-year follow-up by MDS category for selected one-year birth cohorts in men (pooled sample, model 2, birth cohorts defined by age at baseline).

**Fig 2:**
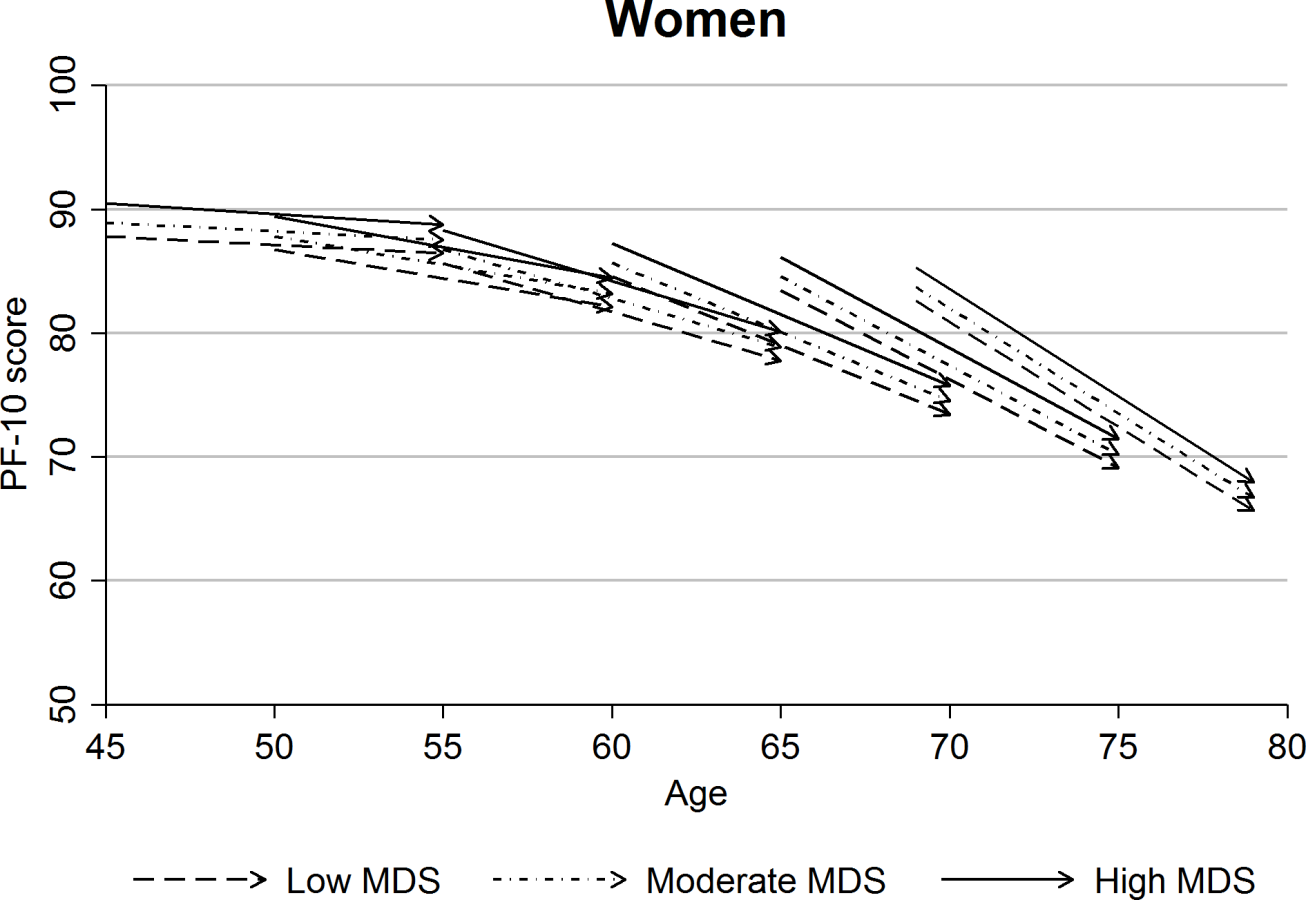
Ageing-vector graphs of predicted initial status and slope of PF-10 score during the 10-year follow-up by MDS category for selected one-year birth cohorts in women (pooled sample, model 2, birth cohorts defined by age at baseline).

Tables 3 and 4 show the associations of MDS with baseline PF-10 scores (initial status) and the rate of its decline (slope). At baseline, participants with higher adherence to the Mediterranean diet scored higher in the PF-10 scale even after multivariable adjustment. With the exception of Czech males (p = 0.20), the results were statistically significant in all country cohorts and in both genders (p<0.01), although the differences in PF-10 score between participants with high and low MDS were small: the 2.15-point (95% CI: 1.31; 2.98) and 2.67-point (1.80; 3.55) difference between the highest and lowest MDS categories in the pooled samples for males and females were equivalent to an age effect of 1.45 (0.88; 2.01) years and 1.73 (1.17; 2.30) years, respectively. When the relationship with the longitudinal PF decline was assessed, we found no statistically significant link between MDS and the rate of decline in any of the examined samples. Although most between-country variations in intercepts and slopes of decline were small, the formal heterogeneity tests were statistically significant in both males and females (p<0.001), reflecting the large sample size rather than clinically meaningful differences. Therefore, we consider pooled results from all three countries shown in Figs 1 and 2 a sensible summary of the overall findings. The figures, showing the multivariable adjusted results in the pooled cohorts using ageing-vector graphs, illustrate that the differences in PF-10 score between the three MDS categories did not diverge over the 10-year follow up.

**Table 3:** Associations of Mediterranean diet score (MDS) with physical functioning trajectories in men.

Cohort	MDS Category	Initial status				Slope			
		Model 1		Model 2		Model 1		Model 2	
		Coefficient^a^ (95% CI)	p-value	Coefficient^a^ (95% CI)	p-value	Coefficient^b^ (95% CI)	p-value	Coefficient^b^ (95% CI)	p-value
CZECH REPUBLIC	MDS low (1–7)	Ref.		Ref.		Ref.		Ref.	
	MDS moderate (8–10)	0.62 (-0.69, 1.93)	0.35	0.95 (-0.25, 2.15)	0.12	-0.11 (-0.31, 0.08)	0.25	-0.12 (-0.31, 0.07)	0.21
	MDS high (11–16)	0.28 (-1.29, 1.85)	0.73	0.78 (-0.63, 2.18)	0.28	-0.04 (-0.27, 0.18)	0.72	-0.08 (-0.30, 0.15)	0.52
	Continuous MDS^c^	0.07 (-0.18, 0.33)	0.57	0.15 (-0.08, 0.38)	0.20	0.00 (-0.04, 0.03)	0.87	-0.01 (-0.04, 0.03)	0.65
RUSSIA	MDS low (1–7)	Ref.		Ref.		Ref.		Ref.	
	MDS moderate (8–10)	0.87 (-0.31, 2.06)	0.15	0.84 (-0.26, 1.94)	0.14	0.02 (-0.22, 0.27)	0.86	-0.02 (-0.26, 0.23)	0.90
	MDS high (11–16)	3.62 (1.99, 5.25)	<0.01	2.84 (1.31, 4.37)	<0.01	0.03 (-0.31, 0.37)	0.88	-0.09 (-0.43, 0.25)	0.59
	Continuous MDS^c^	0.55 (0.29, 0.81)	<0.01	0.43 (0.18, 0.67)	<0.01	0.00 (-0.05, 0.06)	0.88	-0.02 (-0.07, 0.04)	0.56
POLAND	MDS low (1–7)	Ref.		Ref.		Ref.		Ref.	
	MDS moderate (8–10)	1.93 (0.42, 3.44)	0.01	1.64 (0.54, 3.04)	0.02	-0.10 (-0.34, 0.14)	0.41	-0.13 (-0.37, 0.11)	0.30
	MDS high (11–16)	3.58 (2.00, 5.16)	<0.01	2.77 (1.29, 4.24)	<0.01	-0.20 (-0.47, 0.07)	0.15	-0.24 (-0.51, 0.02)	0.07
	Continuous MDS^c^	0.64 (0.39, 0.90)	<0.01	0.52 (0.28, 0.76)	<0.01	-0.03 (-0.07, 0.01)	0.19	-0.04 (-0.08, 0.01)	0.10
POOLED	MDS low (1–7)	Ref.		Ref.		Ref.		Ref.	
	MDS moderate (8–10)	1.12 (0.35, 1.88)	<0.01	1.13 (0.41, 1.84)	<0.01	-0.04 (-0.18, 0.09)	0.52	-0.07 (-0.21, 0.06)	0.29
	MDS high (11–16)	2.47 (1.56, 3.37)	<0.01	2.15 (1.31, 2.98)	<0.01	-0.08 (-0.23, 0.07)	0.31	-0.14 (-0.29, 0.01)	0.07
	Continuous MDS^c^	0.44 (0.29, 0.59)	<0.01	0.39 (0.25, 0.52)	<0.01	-0.01 (-0.03, 0.01)	0.37	-0.02 (-0.04, 0.00)	0.09

^a^ Coefficients for the “initial status” show the difference in mean PF-10 score at baseline between the respective categories and the reference category.

^b^ Coefficients for the “slope” indicate the difference in the mean annual PF-10 score change between the respective categories and the reference category.

^c^ Per 1-unit increase (centered on the value 9)

Model 1: adjusted for baseline age centred at 58 years (and country cohort in case of the pooled sample)

Model 2: adjusted for baseline age centred at 58 years, smoking, marital status, education, ownership of household items, economic activity, joint/spine problem (and country cohort in case of the pooled sample)

**Table 4:** Associations of Mediterranean diet score (MDS) with physical functioning trajectories in women.

Cohort	MDS Category	Initial status				Slope			
		Model 1		Model 2		Model 1		Model 2	
		Coefficient^a^ (95% CI)	p-value	Coefficient^a^ (95% CI)	p-value	Coefficient^b^ (95% CI)	p-value	Coefficient^b^ (95% CI)	p-value
CZECH REPUBLIC	MDS low (1–7)	Ref.		Ref.		Ref.		Ref.	
	MDS moderate (8–10)	0.81 (-0.71, 2.34)	0.30	0.67 (-0.70, 2.04)	0.34	0.13 (-0.09, 0.35)	0.24	0.11 (-0.10, 0.33)	0.31
	MDS high (11–16)	2.65 (1.08, 4.21)	<0.01	2.14 (0.72, 3.57)	<0.01	0.18 (-0.03, 0.39)	0.10	0.15 (-0.07, 0.36)	0.17
	Continuous MDS^c^	0.52 (0.28, 0.76)	<0.01	0.41 (0.19, 0.64)	<0.01	0.03 (0.00, 0.06)	0.09	0.02 (-0.01, 0.06)	0.16
RUSSIA	MDS low (1–7)	Ref.		Ref.		Ref.		Ref.	
	MDS moderate (8–10)	0.68 (-0.55, 1.91)	0.28	0.48 (-0.70, 1.66)	0.43	0.07 (-0.15, 0.29)	0.52	0.05 (-0.17, 0.27)	0.68
	MDS high (11–16)	2.65 (0.99, 4.31)	<0.01	2.02 (0.41, 3.62)	0.01	0.08 (-0.22, 0.38)	0.60	0.00 (-0.30, 0.30)	0.99
	Continuous MDS^c^	0.51 (0.25, 0.78)	<0.01	0.38 (0.12, 0.64)	<0.01	0.00 (-0.04, 0.05)	0.90	-0.01 (-0.06, 0.04)	0.61
POLAND	MDS low (1–7)	Ref.		Ref.		Ref.		Ref.	
	MDS moderate (8–10)	2.47 (0.88, 4.07)	<0.01	2.25 (0.76, 3.74)	<0.01	-0.15 (-0.42, 0.12)	0.27	-0.17 (-0.44, 0.10)	0.21
	MDS high (11–16)	4.09 (2.39, 5.79)	<0.01	3.62 (2.02, 5.23)	<0.01	-0.17 (-0.45, 0.12)	0.25	-0.23 (-0.51, 0.06)	0.12
	Continuous MDS^c^	0.68 (0.42, 0.95)	<0.01	0.62 (0.37, 0.88)	<0.01	-0.02 (-0.06, 0.03)	0.51	-0.03 (-0.07, 0.02)	0.24
POOLED	MDS low (1–7)	Ref.		Ref.		Ref.		Ref.	
	MDS moderate (8–10)	1.24 (0.42, 2.06)	<0.01	1.10 (0.33, 1.87)	<0.01	0.02 (-0.13, 0.16)	0.83	0.00 (-0.14, 0.14)	0.98
	MDS high (11–16)	3.08 (2.15, 4.01)	<0.01	2.67 (1.80, 3.55)	<0.01	0.02 (-0.13, 0.17)	0.80	-0.03 (-0.19, 0.12)	0.67
	Continuous MDS^c^	0.58 (0.43, 0.73)	<0.01	0.50 (0.36, 0.64)	<0.01	0.00 (-0.02, 0.03)	0.73	-0.01 (-0.03, 0.02)	0.65

^a^ Coefficients for the “initial status” show the difference in mean PF-10 score at baseline between the respective categories and the reference category.

^b^ Coefficients for the “slope” indicate the difference in the mean annual PF-10 score change between the respective categories and the reference category.

^c^ Per 1-unit increase (centered on the value 9)

Model 1: adjusted for baseline age centred at 58 years (and country cohort in case of the pooled sample)

Model 2: adjusted for baseline age centred at 58 years, smoking, marital status, education, ownership of household items, economic activity, joint/spine problem (and country cohort in case of the pooled sample)

Sensitivity analyses showed that further adjustment for BMI and physical activity reduced the strength of the association in most subgroups, however it did not materially change the direction and significance of the main findings (S1 and S2 Tables). Furthermore, results were similar when the analysis was stratified into two groups by mean BMI or by median of baseline PF-10 score (S3 and S4 Tables).

## Discussion

### Main findings

This large study of three Eastern European population based cohorts examined the association between Mediterranean dietary pattern and trajectories of physical functioning over a 10-year period. We found better physical functioning in those with higher adherence to the Mediterranean diet at baseline, albeit the difference in PF-10 score between high and low MDS categories was modest. No association was observed between MDS and the rate of physical decline over time.

### Interpretation

The literature on the association between Mediterranean diet and physical functioning is not extensive. Previous longitudinal analyses which examined trajectories in physical decline suggested that high adherence to Mediterranean diet was significantly related to slower reduction in walking speed and lower body mobility [17–19]. A few cross-sectional and cohort studies have also found a moderately strong inverse association of the Mediterranean dietary pattern with reduced physical functioning and risk of disability, however, the evidence is not entirely consistent [20,41–43]. While the cross-sectional results of our analysis are similar to those reported in the literature, the results regarding the rate of PF decline by MDS are inconsistent with studies which had reported slower decline in individuals with better diet.

There are a number of factors which can explain the nil-findings of the longitudinal analysis. Firstly, there is a possibility that measurement error of dietary intakes reduced the capacity of the data to identify actually existing associations. However, the fact that the cross-sectional results pointed to the expected direction and that previous analysis showed strong associations between the same MDS and mortality outcomes [27] makes this possibility less likely.

Secondly, the measurement error might have affected the PF-10 scores as well, which in turn, could have an impact on the effect estimates. While some previous studies used objective measures of physical performance, the current analysis was based on self-report. However, we have showed earlier that the PF-10 score agreed well with objective measurements of chair rise and grip strength in the HAPIEE study, suggesting adequate validity of the PF-10 data [44]. Notwithstanding this, self-reported questionnaires provide a subjective assessment of functioning relative to age and sex-peers as well as personal expectations; thus some differences could also be expected between associations based on objective and subjective functional evaluations.

Thirdly, the modest benefits of a greater adherence to a Mediterranean diet on physical functioning observed at baseline may reflect healthier diet throughout the lifetime, prior to the baseline. While the difference in physical functioning across the MDS categories was maintained during the 10-year observational period, it is possible that a longer follow-up would be needed in this sample to detect significant divergence in the trajectories.

Fourth, there is a possibility that, in contrast to findings of previous studies, diet quality might not actually affect the rate of physical decline of aging populations in Eastern Europe. It has been suggested that alcohol consumption and the prevalence of tobacco smoking in Eastern Europe is high [45,46], and it is likely that these lifestyle factors have a strong relationship with physical functioning [47]. In fact, in a previous analysis we found that alcohol consumption was related to faster physical decline over time in several country-cohorts of the HAPIEE study [44]. Further studies should estimate the extent of which the different lifestyle factors contribute to the decline of physical functioning in older Eastern Europeans. In addition to lifestyle factors, our sample may also differ from other populations in terms of the effect of socio-economic characteristics on physical functioning, which seems to be weaker here than in previous studies.

Finally, the age range of participants in our analysis was somewhat lower than most previous studies (45–70 vs 65+), which could also affect the longitudinal trends. However, the fact that we found parallel trajectories in analyses restricted to the older age groups (i.e. 65+) suggests that this issue was unlikely to be the primary reason for the lack of longitudinal associations between MDS and physical functioning.

### Limitations and strengths

In addition to the previously mentioned important issue of measurement error, there are several other potential limitations which need to be acknowledged. Most importantly, due to the moderate response rate (61%) and the fact that recruitment to the HAPIEE study took place only in urban areas, our findings are not fully representative of whole population of Russia, Poland and the Czech Republic. It is likely that the cohorts are healthier than the general population; this however, should not affect the internal validity of the findings.

Second, similarly to other observational studies we cannot entirely exclude the possibility of residual confounding. However, the fact that the associations were adjusted for a wide range of socio-economic and lifestyle factors and used overall dietary pattern as the exposure variable, as opposed to individual nutrients or foods, significantly reduced the room for potential confounding.

Further limitation is the fact that dietary intakes were measured only at baseline and we have no information on whether participants maintained their respective diet over time. As there is some evidence for dietary pattern changes in Eastern Europe between 1990 and 2010 [48], repeated nutritional assessments could strengthen the analysis in future studies.

On the other hand, the study has important strengths. This is one of the largest cohort study which investigated the relationship between overall diet and trajectories of physical functioning in aging general population samples. This large sample size improves the statistical power and reliability of the finding. That fact that the study was based in Eastern Europe is also important, as studying determinants of ageing outcomes in different social settings addresses the issue of generalizability of observed associations.

## Conclusion

Our results do not support the hypothesis that adherence to the Mediterranean diet influences the slope of physical decline in older age. On the other hand, the modest differences in physical functioning between Mediterranean diet groups may reflect long-term dietary habits prior to baseline. Further studies with longer follow-up are needed to investigate the role of the Mediterranean diet and population-specific dietary patterns on physical functioning.

## Supporting information

S1 Fig PF-10 score trajectories during the 10-year follow-up by country for selected one-year birth cohort by country in men (empty model, birth cohorts defined by age at baseline). (PDF)

S2 Fig PF-10 score trajectories during the 10-year follow-up for every fifth one-year birth cohort by country in women (empty model, birth cohorts defined by age at baseline). (PDF)

S1 Table Associations of Mediterranean diet score (MDS) with physical functioning after further adjustment for physical activity and BMI in males. (DOCX)

S2 Table Associations of Mediterranean diet score (MDS) with physical functioning trajectories after further adjustment for physical activity and BMI in females. (DOCX)

S3 Table Associations of Mediterranean diet score (MDS) with physical functioning trajectories stratified by BMI. (DOCX)

S4 Table Associations of Mediterranean diet score (MDS) with physical functioning trajectories stratified by baseline PF-10 score. (DOCX)
