# Supplementary figures and images for: Mediterranean diet and physical functioning trajectories in Eastern Europe: Findings from the HAPIEE study
Source: PLoS One. 2018 Jul 12;13(7):e0200460. doi: 10.1371/journal.pone.0200460 (PMC6042732; doi:10.1371/journal.pone.0200460)

# Men

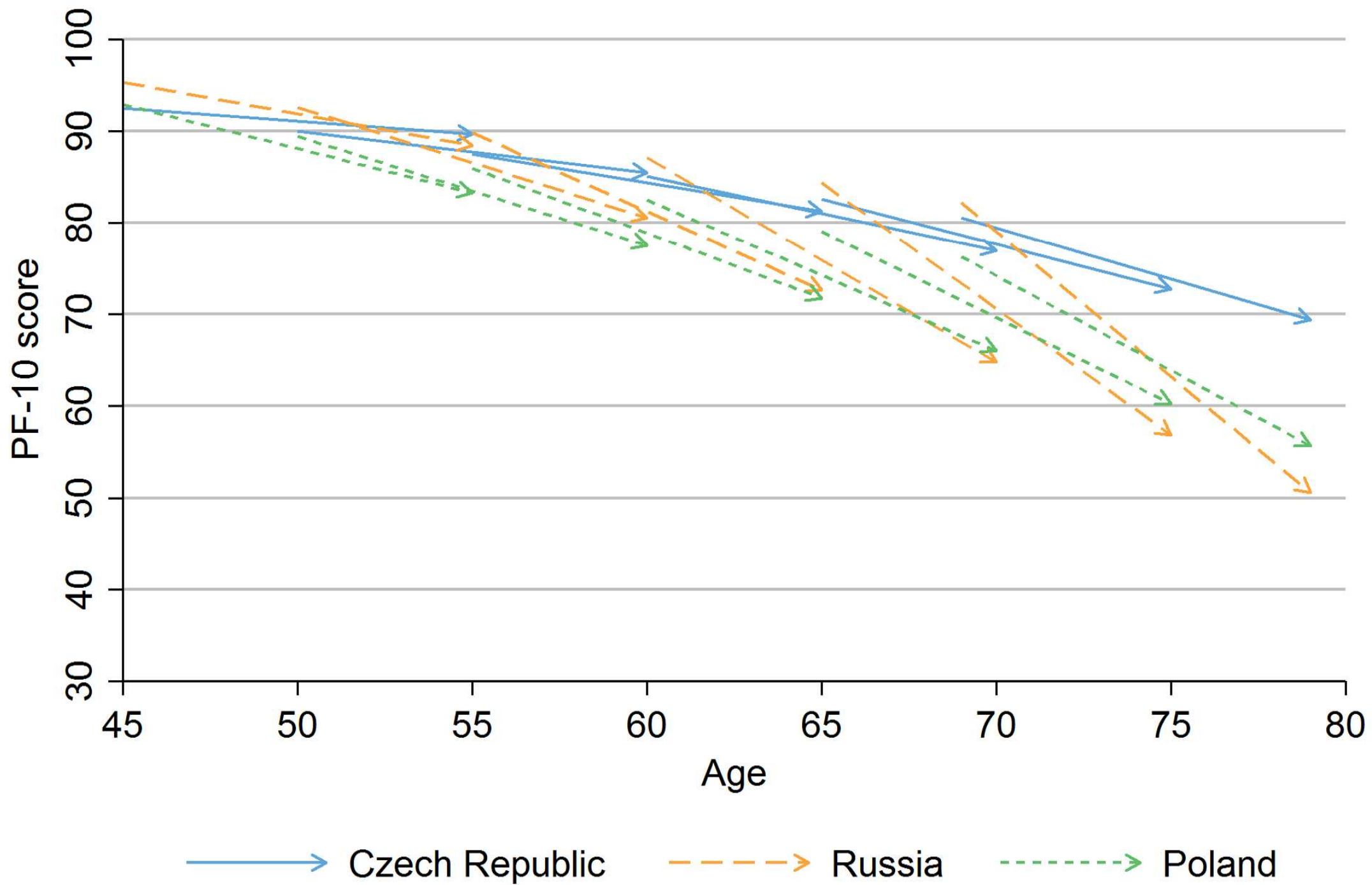

Supplement: S1 Fig — (PDF) [file pone.0200460.s001.pdf]

# Women

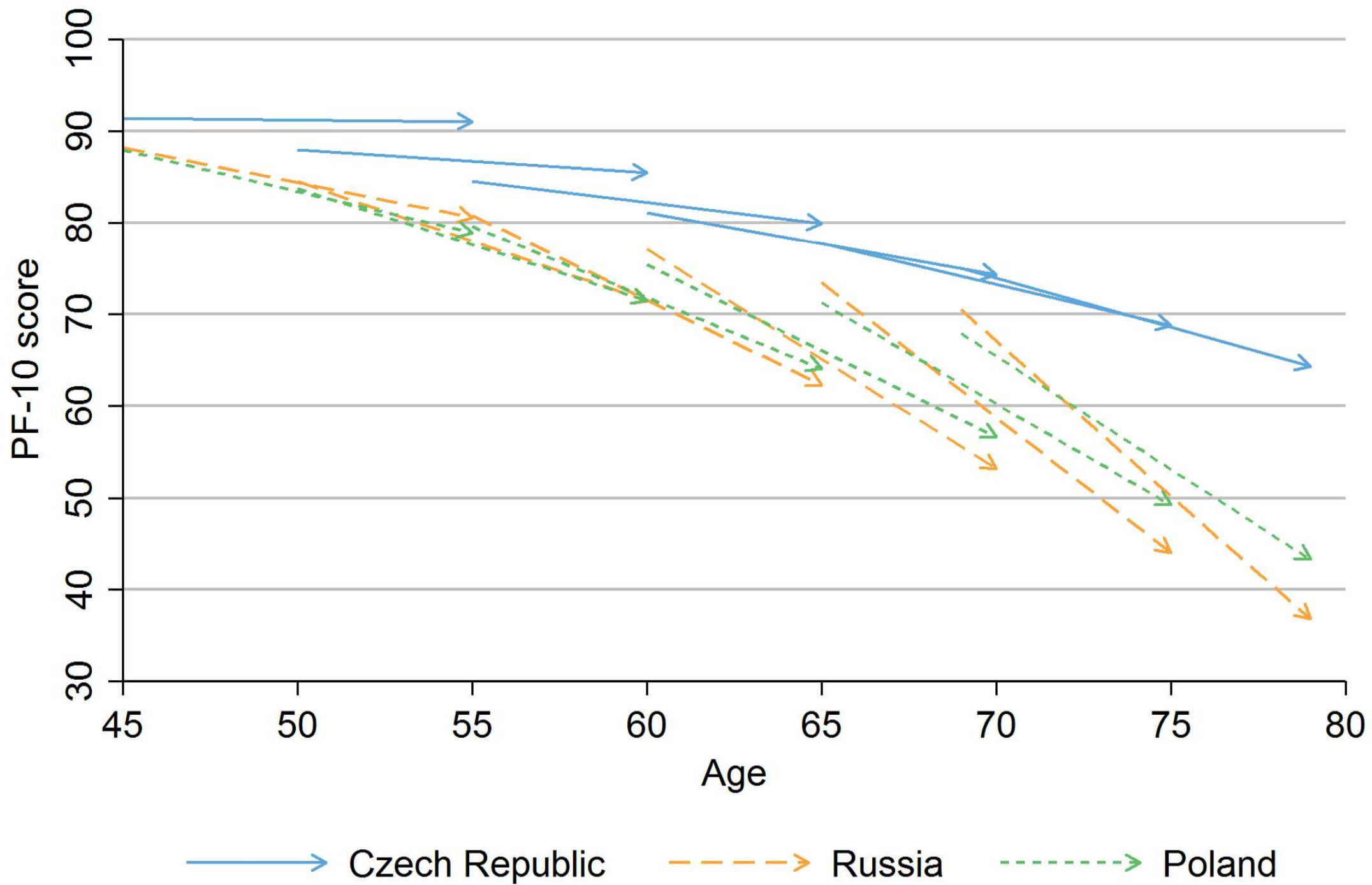

Supplement: S2 Fig — (PDF) [file pone.0200460.s002.pdf]
