# Supplementary material for: Mediterranean diet and physical functioning trajectories in Eastern Europe: Findings from the HAPIEE study
Source: PLoS One. 2018 Jul 12;13(7):e0200460. doi: 10.1371/journal.pone.0200460 (PMC6042732; doi:10.1371/journal.pone.0200460)
Supplement: S1 Table — (DOCX) [file pone.0200460.s003.docx]

**S1 table.** Associations of Mediterranean diet score (MDS) with physical functioning after further adjustment for physical activity and BMI in males

| Cohort | MDS category | Initial status | | Slope | |
| --- | --- | --- | --- | --- | --- |
|  |  | Coefficient (95% CI)^a^ | p-value | Coefficient (95% CI)^b^ | p-value |
| CZECH REPUBLIC | MDS low (1-7) | Ref. |  | Ref. |  |
|  | MDS moderate (8-10) | 0.65 (-0.51, 1.81) | 0.28 | -0.13 (-0.32, 0.06) | 0.19 |
|  | MDS high (11-16) | 0.40 (-0.96, 1.76) | 0.56 | -0.06 (-0.29, 0.16) | 0.59 |
|  | Continuous MDS^c^ | 0.08 (-0.15, 0.30) | 0.50 | -0.01 (-0.04, 0.03) | 0.69 |
| RUSSIA | MDS low (1-7) | Ref. |  | Ref. |  |
|  | MDS moderate (8-10) | 0.92 (-0.14, 1.98) | 0.09 | -0.03 (-0.28, 0.21) | 0.79 |
|  | MDS high (11-16) | 3.01 (1.55, 4.47) | <0.01 | -0.12 (-0.46, 0.22) | 0.48 |
|  | Continuous MDS^c^ | 0.47 (0.24, 0.70) | <0.01 | -0.02 (-0.07, 0.03) | 0.49 |
| POLAND | MDS low (1-7) | Ref. |  | Ref. |  |
|  | MDS moderate (8-10) | 0.91 (-0.43, 2.24) | 0.19 | -0.09 (-0.32, 0.15) | 0.47 |
|  | MDS high (11-16) | 1.52 (0.10, 2.95) | 0.04 | -0.17 (-0.43, 0.10) | 0.22 |
|  | Continuous MDS^c^ | 0.31 (0.08, 0.54) | <0.01 | -0.02 (-0.06, 0.02) | 0.28 |
| POOLED | MDS low (1-7) | Ref. |  | Ref. |  |
|  | MDS moderate (8-10) | 0.88 (0.20, 1.57) | 0.01 | -0.07 (-0.21, 0.06) | 0.29 |
|  | MDS high (11-16) | 1.66 (0.86, 2.46) | <0.01 | -0.13 (-0.28, 0.02) | 0.10 |
|  | Continuous MDS^c^ | 0.31 (0.17, 0.44) | <0.01 | -0.02 (-0.04, 0.00) | 0.11 |

^a^ Coefficients for the “initial status” show the difference in mean PF-10 score at baseline between the respective categories and the reference category.

^b^ Coefficients for the “slope” indicate the difference in the mean annual PF-10 score change between the respective categories and the reference category.

^c^ Per 1-unit increase (centered on the value 9)

All coefficients were adjusted for baseline age centred at 58 years, smoking marital status, education, ownership of household items, economic activity, joint/spine problem, physical activity, BMI (and country cohort in case of the pooled sample)
