# Supplementary material for: Mediterranean diet and physical functioning trajectories in Eastern Europe: Findings from the HAPIEE study
Source: PLoS One. 2018 Jul 12;13(7):e0200460. doi: 10.1371/journal.pone.0200460 (PMC6042732; doi:10.1371/journal.pone.0200460)
Supplement: S2 Table — (DOCX) [file pone.0200460.s004.docx]

**S2 table.** Associations of Mediterranean diet score (MDS) with physical functioning trajectories after further adjustment for physical activity and BMI in females

| Cohort | MDS category | Initial status | | Slope | |
| --- | --- | --- | --- | --- | --- |
|  |  | Coefficient (95% CI)^a^ | p-value | Coefficient (95% CI)^b^ | p-value |
| CZECH REPUBLIC | MDS low (1-7) | Ref. |  | Ref. |  |
|  | MDS moderate (8-10) | 0.47 (-1.87, 1.21) | 0.49 | 0.11 (-0.11, 0.32) | 0.34 |
|  | MDS high (11-16) | 1.43 (0.05, 2.81) | 0.04 | 0.13 (-0.09, 0.34) | 0.25 |
|  | Continuous MDS^c^ | 0.30 (0.08, 0.51) | <0.01 | 0.02 (-0.01, 0.05) | 0.23 |
| RUSSIA | MDS low (1-7) | Ref. |  | Ref. |  |
|  | MDS moderate (8-10) | 0.37 (-0.77, 1.50) | 0.53 | 0.05 (-0.17, 0.27) | 0.66 |
|  | MDS high (11-16) | 1.90 (0.36, 3.45) | 0.02 | 0.00 (-0.30, 0.29) | 0.98 |
|  | Continuous MDS^c^ | 0.34 (0.09, 0.59) | <0.01 | -0.01 (-0.06, 0.03) | 0.58 |
| POLAND | MDS low (1-7) | Ref. |  | Ref. |  |
|  | MDS moderate (8-10) | 1.60 (0.14, 3.05) | 0.03 | -0.14 (-0.41, 0.13) | 0.30 |
|  | MDS high (11-16) | 2.67 (1.10, 4.23) | <0.01 | -0.17 (-0.45, 0.11) | 0.24 |
|  | Continuous MDS^c^ | 0.49 (0.24, 0.74) | <0.01 | -0.02 (-0.06, 0.03) | 0.42 |
| POOLED | MDS low (1-7) | Ref. |  | Ref. |  |
|  | MDS moderate (8-10) | 0.84 (0.09, 1.59) | 0.03 | 0.00 (-0.15, 0.14) | 0.96 |
|  | MDS high (11-16) | 2.11 (1.25, 2.96) | <0.01 | -0.03 (-0.19, 0.12) | 0.66 |
|  | Continuous MDS^c^ | 0.41 (0.27, 0.55) | <0.01 | -0.01 (-0.03, 0.02) | 0.62 |

^a^ Coefficients for the “initial status” show the difference in mean PF-10 score at baseline between the respective categories and the reference category.

^b^ Coefficients for the “slope” indicate the difference in the mean annual PF-10 score change between the respective categories and the reference category.

^c^ Per 1-unit increase (centered on the value 9)

All coefficients were adjusted for baseline age centred at 58 years, marital status, education, ownership of household items, economic activity, joint/spine problem, smoking status, physical activity, BMI (and country cohort in case of the pooled sample)
