# Supplementary material for: Mediterranean diet and physical functioning trajectories in Eastern Europe: Findings from the HAPIEE study
Source: PLoS One. 2018 Jul 12;13(7):e0200460. doi: 10.1371/journal.pone.0200460 (PMC6042732; doi:10.1371/journal.pone.0200460)
Supplement: S3 Table — (DOCX) [file pone.0200460.s005.docx]

**S3 table.** Associations of Mediterranean diet score (MDS) with physical functioning trajectories stratified by BMI

| Sex | BMI category^a^ | MDS Category | **Initial status** | | | | | **Slope** | | | | |
| --- | --- | --- | --- | --- | --- | --- | --- | --- | --- | --- | --- | --- |
|  |  |  | Model 1 | |  | Model 2 | | Model 1 | |  | Model 2 | |
|  |  |  | Coefficient (95% CI)^b^ | p-value |  | Coefficient (95% CI)^b^ | p-value | Coefficient (95% CI)^c^ | p-value |  | Coefficient (95% CI)^c^ | p-value |
| MALES | BMI high | MDS low (1-7) | Ref. |  |  | Ref. |  | Ref. |  |  | Ref. |  |
|  |  | MDS moderate (8-10) | 0.85 (-0.36, 2.07) | 0.17 |  | 0.97 (-0.17, 2.10) | 0.10 | 0.00 (-0.21, 0.20) | 0.97 |  | -0.02 (-0.23, 0.18) | 0.82 |
|  |  | MDS high (11-16) | 2.14 (0.70, 3.57) | <0.01 |  | 1.93 (0.62, 3.24) | <0.01 | -0.09 (-0.32, 0.15) | 0.47 |  | -0.13 (-0.36, 0.10) | 0.28 |
|  |  | Continuous MDS^d^ | 0.39 (0.16, 0.63) | <0.01 |  | 0.37 (0.15, 0.59) | <0.01 | -0.02 (-0.06, 0.02) | 0.33 |  | -0.03 (-0.06, 0.01) | 0.17 |
|  | BMI low | MDS low (1-7) | Ref. |  |  | Ref. |  | Ref. |  |  | Ref. |  |
|  |  | MDS moderate (8-10) | 1.43 (0.41, 2.44) | <0.01 |  | 1.28 (0.34, 2.23) | <0.01 | -0.06 (-0.24, 0.13) | 0.47 |  | -0.12 (-0.31, 0.07) | 0.22 |
|  |  | MDS high (11-16) | 2.96 (1.72, 4.21) | <0.01 |  | 2.47 (1.31, 3.62) | <0.01 | -0.07 (-0.28, 0.15) | 0.68 |  | -0.14 (-0.35, 0.07) | 0.30 |
|  |  | Continuous MDS^d^ | 0.50 (0.30, 0.70) | <0.01 |  | 0.42 (0.23, 0.60) | <0.01 | 0.00 (-0.04, 0.03) | 0.93 |  | -0.02 (-0.05, 0.02) | 0.33 |
|  |  |  |  |  |  |  |  |  |  |  |  |  |
| FEMALES | BMI high | MDS low (1-7) | Ref. |  |  | Ref. |  | Ref. |  |  | Ref. |  |
|  |  | MDS moderate (8-10) | 0.67 (-0.62, 1.96) | 0.31 |  | 0.43 (-0.78, 1.65) | 0.49 | 0.05 (-0.16, 0.25) | 0.68 |  | 0.02 (-0.20, 0.25) | 0.84 |
|  |  | MDS high (11-16) | 2.08 (0.52, 3.63) | <0.01 |  | 1.50 (0.03, 2.97) | 0.04 | -0.02 (-0.27, 0.23) | 0.89 |  | -0.08 (-0.33, 0.17) | 0.53 |
|  |  | Continuous MDS^d^ | 0.46 (0.20, 0.71) | <0.01 |  | 0.35 (0.11, 0.59) | <0.01 | -0.01 (-0.05, 0.03) | 0.68 |  | -0.02 (-0.06, 0.02) | 0.35 |
|  | BMI low | MDS low (1-7) | Ref. |  |  | Ref. |  | Ref. |  |  | Ref. |  |
|  |  | MDS moderate (8-10) | 1.47 (0.40, 2.53) | <0.01 |  | 1.46 (0.47, 2.45) | <0.01 | -0.03 (-0.21, 0.15) | 0.76 |  | -0.04 (-0.22, 0.14) | 0.66 |
|  |  | MDS high (11-16) | 3.50 (2.34, 4.65) | <0.01 |  | 3.28 (2.20, 4.36) | <0.01 | 0.03 (-0.17, 0.22) | 0.78 |  | -0.02 (-0.21, 0.17) | 0.84 |
|  |  | Continuous MDS^d^ | 0.59 (0.41, 0.77) | <0.01 |  | 0.55 (0.37, 0.72) | <0.01 | 0.01 (-0.02, 0.04) | 0.46 |  | 0.00 (-0.03, 0.03) | 0.90 |

^a^ BMI groups were stratified by sex-specific means (men: 27.6 kg/m2; women: 29.0 kg/m2)

^b^ Coefficients for the “initial status” show the difference in mean PF-10 score at baseline between the respective categories and the reference category.

^c^ Coefficients for the “slope” indicate the difference in the mean annual PF-10 score change between the respective categories and the reference category.

^d^ Per 1-unit increase (centered on the value 9)

Model 1: adjusted for baseline age centred at 58 years (and country cohort in case of the pooled sample)

Model 2: adjusted for baseline age centred at 58 years, smoking, marital status, education, ownership of household items, economic activity, joint/spine problem (and country cohort in case of the pooled sample)
