# Supplementary material for: Mediterranean diet and physical functioning trajectories in Eastern Europe: Findings from the HAPIEE study
Source: PLoS One. 2018 Jul 12;13(7):e0200460. doi: 10.1371/journal.pone.0200460 (PMC6042732; doi:10.1371/journal.pone.0200460)
Supplement: S4 Table — (DOCX) [file pone.0200460.s006.docx]

**S4 table.** Associations of Mediterranean diet score (MDS) with physical functioning trajectories stratified by baseline PF-10 score

| Sex | PF-10 score category^a^ | MDS Category | **Initial status** | | | | | **Slope** | | | | |
| --- | --- | --- | --- | --- | --- | --- | --- | --- | --- | --- | --- | --- |
|  |  |  | Model 1 | |  | Model 2 | | Model 1 | |  | Model 2 | |
|  |  |  | Coefficient  (95% CI)^b^ | p-value |  | Coefficient  (95% CI)^b^ | p-value | Coefficient  (95% CI)^c^ | p-value |  | Coefficient  (95% CI)^c^ | p-value |
| MALES | PF-10 high | MDS low (1-7) | Ref. |  |  | Ref. |  | Ref. |  |  | Ref. |  |
|  |  | MDS moderate (8-10) | 0.39 (0.14, 0.64) | <0.01 |  | 0.41 (0.07, 0.75) | 0.02 | 0.05 (-0.10, 0.21) | 0.51 |  | 0.02 (-0.19, 0.20) | 0.84 |
|  |  | MDS high (11-16) | 0.61 (0.32, 0.91) | <0.01 |  | 0.62 (0.22, 1.02) | <0.01 | -0.01 (-0.19, 0.18) | 0.93 |  | -0.07 (-0.29, 0.14) | 0.50 |
|  |  | Continuous MDS^d^ | 0.12 (0.07, 0.16) | <0.01 |  | 0.12 (0.04, 0.19) | <0.01 | 0.00 (-0.03, 0.03) | 0.78 |  | -0.02 (-0.05, 0.02) | 0.39 |
|  | PF-10 low | MDS low (1-7) | Ref. |  |  | Ref. |  | Ref. |  |  | Ref. |  |
|  |  | MDS moderate (8-10) | 0.68 (-0.77, 2.13) | 0.36 |  | 0.83 (-0.55, 2.20) | 0.24 | -0.08 (-0.34, 0.19) | 0.57 |  | -0.09 (-0.35, 0.17) | 0.51 |
|  |  | MDS high (11-16) | 1.59 (-0.19, 3.36) | 0.08 |  | 1.52 (-0.17, 3.20) | 0.08 | -0.08 (-0.39, 0.24) | 0.62 |  | -0.10 (-0.42, 0.22) | 0.55 |
|  |  | Continuous MDS^d^ | 0.29 (0.00, 0.58) | 0.05 |  | 0.30 (0.03, 0.57) | 0.03 | 0.00 (-0.05, 0.05) | 0.89 |  | -0.01 (-0.06, 0.04) | 0.75 |
|  |  |  |  |  |  |  |  |  |  |  |  |  |
| FEMALES | PF-10 high | MDS low (1-7) | Ref. |  |  | Ref. |  | Ref. |  |  | Ref. |  |
|  |  | MDS moderate (8-10) | 0.45 (0.06, 0.83) | 0.02 |  | 0.47 (0.08, 0.87) | 0.02 | 0.07 (-0.08, 0.23) | 0.36 |  | 0.06 (-0.10, 0.21) | 0.46 |
|  |  | MDS high (11-16) | 0.78 (0.36, 1.21) | <0.01 |  | 0.87 (0.38, 1.36) | <0.01 | 0.10 (-0.07, 0.27) | 0.24 |  | 0.06 (-0.11, 0.23) | 0.48 |
|  |  | Continuous MDS^d^ | 0.11 (0.05, 0.18) | <0.01 |  | 0.12 (0.05, 0.19) | <0.01 | 0.03 (0.00, 0.05) | 0.07 |  | 0.02 (-0.01, 0.04) | 0.21 |
|  | PF-10 low | MDS low (1-7) | Ref. |  |  | Ref. |  | Ref. |  |  | Ref. |  |
|  |  | MDS moderate (8-10) | 0.94 (-0.24, 2.12) | 0.12 |  | 0.79 (-0.35, 1.93) | 0.17 | 0.01 (-0.20, 0.23) | 0.90 |  | 0.00 (-0.22, 0.21) | 0.99 |
|  |  | MDS high (11-16) | 1.67 (0.27, 3.08) | 0.02 |  | 1.28 (-0.09, 2.65) | 0.07 | 0.07 (-0.19, 0.33) | 0.59 |  | 0.01 (-0.25, 0.27) | 0.93 |
|  |  | Continuous MDS^d^ | 0.37 (0.14, 0.60) | <0.01 |  | 0.31 (0.09, 0.53) | <0.01 | 0.01 (-0.04, 0.05) | 0.82 |  | -0.01 (-0.05, 0.04) | 0.81 |

^a^ BMI groups were stratified by sex-specific means (men: 27.6 kg/m2; women: 29.0 kg/m2)

^b^ Coefficients for the “initial status” show the difference in mean PF-10 score at baseline between the respective categories and the reference category.

^c^ Coefficients for the “slope” indicate the difference in the mean annual PF-10 score change between the respective categories and the reference category.

^d^ Per 1-unit increase (centered on the value 9)

Model 1: adjusted for baseline age centred at 58 years (and country cohort in case of the pooled sample)

Model 2: adjusted for baseline age centred at 58 years, smoking, marital status, education, ownership of household items, economic activity, joint/spine problem (and country cohort in case of the pooled sample)
